# Pediatric Pancreatic Tuberculosis: A Case Report and Review of the Literature

**DOI:** 10.1155/2018/5215128

**Published:** 2018-08-30

**Authors:** Yajun Zhang, Yuhong Tao

**Affiliations:** ^1^Department of Pediatrics, West China Second University Hospital, Sichuan University, Chengdu, Sichuan, China; ^2^Key Laboratory of Birth Defects and Related Diseases of Women and Children, Sichuan University, Ministry of Education, Chengdu, Sichuan, China

## Abstract

Pancreatic tuberculosis (TB) is an uncommon form of extrapulmonary TB and represents a diagnostic challenge for physicians. Pancreatic TB presents with nonspecific signs and symptoms and may mimic malignancy. However, pancreatic TB rarely occurs in children. Here, we present a case of a 5-year-old girl with pancreatic TB and markedly elevated serum cancer antigen- (CA-) 125 levels, thus raising the suspicion of malignancy, but positivity for *Mycobacterium tuberculosis* DNA was noted. The patient recovered after being administered standard antitubercular therapy for one year. This case suggests that clinicians should have a heightened suspicion of pancreatic TB when faced with pancreatic lesions despite the fact that increased CA-125 may indicate malignancy. Laparoscopy combined with peritoneal biopsy and polymerase chain reaction (PCR) may provide a new method to confirm the diagnosis.

## 1. Introduction

Pancreatic tuberculosis (TB) is extremely rare in either immunocompetent or immunosuppressed hosts, even in regions with a high prevalence of TB [1]. Pancreatic TB has no specific clinical features. Both abdominal CT and MRI have a diagnostic value, but pancreatic TB is easily misdiagnosed as a pancreatic tumor. Pancreatic TB accounts for 2.0–4.7% of autopsied patients with pulmonary TB [2, 3]. It is relatively common in young and middle-aged patients and rarely occurs in children [4]. Here, we present the case of a 5-year-old girl with pancreatic TB.

## 2. Case Presentation

**Figure 1:**
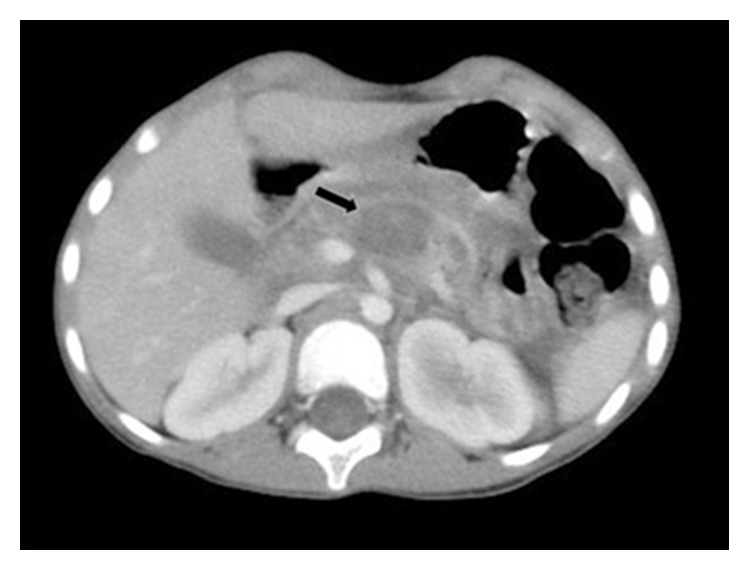
Abdominal enhanced CT showing a cystic low-density shadow (arrow) in the head and neck of the pancreas.

A 5-year-old girl who had no previous medical history was admitted to our hospital with a right groin mass for 2 months and abdominal distension for 15 days. Her symptoms were accompanied by occasional umbilical pain, vomiting, and diarrhea. Physical examination showed acute facial features, malnutrition, abdominal swelling, apparent tenderness, a mass approximately 4 × 3 × 2 cm in size in the right inguinal region, and negative Grey Turner sign and Cullen sign. Routine blood test revealed white blood cells 2.97 × 10^9^/L, neutrophils 47.2%, red blood cells 4.88 × 10^12^/L, platelets 714 × 10^9^/L, and C-reactive protein 2.06 mg/L. Abdominal enhanced computed tomography (CT) revealed the following findings: (1) the pancreatic duct was significantly dilated, and the surrounding pancreatic head space was unclear; (2) a cystic low-density shadow was observed in the head of the pancreas (Figure 1); (3) massive peritoneal effusion was observed; (4) patchy lymph node enhancement and enlargement were observed in the mesentery; and (5) intestinal aggregation in the upper abdomen along with thickening and enhancement of the bowel wall was observed. Chest CT revealed increased lung markings and no signs of TB.

After admission, the patient presented with diffuse abdominal distension. Laboratory tests indicated pancreatitis (serum lipase 3167 U/L and serum amylase 720 U/L). Serum cancer antigen- (CA-) 125 was increased to 484.5 U/mL (normal is less than 35 U/mL). An abdominal puncture was performed three days after admission. Ascites was red and yellow. Qualitative protein was positive. No acid-fast bacilli were identified via ascites smear. The anti-TB antibody in serum was negative. The purified protein derivative of the tuberculin test and interferon-gamma release assay were negative. No tumor cells were identified in the ascites, and there was no bacterial growth in the ascites for 48 hours. Seven days after admission, the patient underwent laparoscopic exploration. In total, 3600 mL of bloody ascites was present in the peritoneal cavity. Pale and fish-like masses were found. A wide and dark-red flocculent area was visible. Pathological examination revealed an inflammatory exudate. The results of periodic acid-Schiff staining, methenamine silver staining, and acid-fast staining were negative. No bacteria or tubercle bacilli were found in the ascites cultures. Using polymerase chain reaction (PCR), *Mycobacterium tuberculosis* DNA was identified in the resected abdominal mass. Therefore, the diagnosis of pancreatic TB was made. Fasting, somatostatin, omeprazole, and total parenteral nutrition therapy were administered. Rifampicin (10 mg/kg/day), pyrazinamide (20 mg/kg/day), and isoniazid (10 mg/kg/day) were prescribed. Three weeks later, abdominal pain, abdominal distension, cough, fever, nausea, vomiting, and diarrhea had resolved. The patient was discharged.

Two weeks after discharge, pain around the umbilicus and periumbilical tenderness were reported with nausea and vomiting. Abdominal ultrasonography revealed large pancreatic cysts. The diagnosis of a pancreatic pseudocyst was made, and ultrasound-guided puncture drainage was performed. Giant cystic lesions as thick as the wall and approximately 12 × 9 × 10 cm in size were observed by intraoperative ultrasonography. Approximately 500 mL of brown cyst fluid was expelled. On day 4 after the operation, the patient's general condition was good, and her vital signs were stable. Therefore, she was discharged again. Outpatient antitubercular therapy consisted of isoniazid, rifampicin, and pyrazinamide for 4 months and subsequently isoniazid and rifampicin for 8 months.

**Figure 2:**
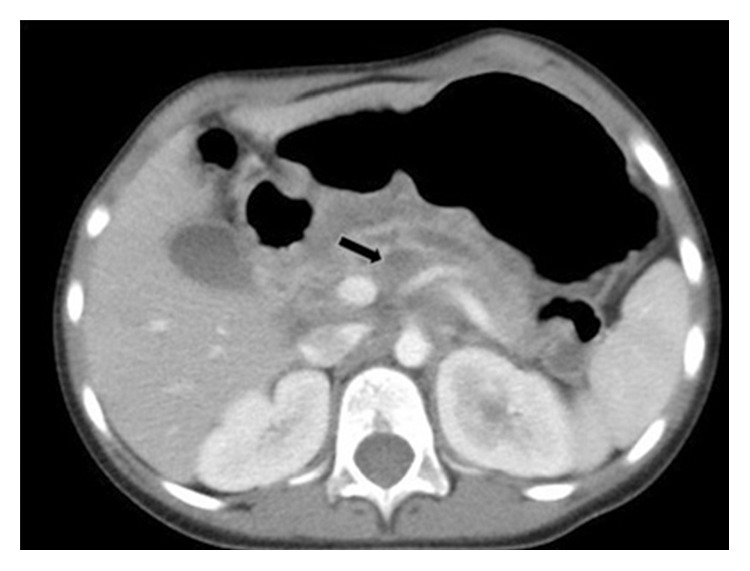
Abdominal enhanced CT showing a significant reduction in the cystic low-density shadow (arrow) of the head and neck of the pancreas one year after discharge.

At the one-year outpatient follow-up visit, her appetite improved, and she regained the weight she had previously lost. Abdominal CT showed a significant reduction in the cystic low-density shadow of the head and neck of the pancreas (Figure 2). The effect of the antitubercular therapy was significant.

## 3. Discussion

To the best of our knowledge, this is the first pediatric case of pancreatic TB. The pancreas is relatively resistant to TB because the pathogen is destroyed by pancreatic enzymes [1, 5]. However, once TB breaks through this line of defense, it may cause pancreatic TB. In recent years, reports of pancreatic TB with antibiotic resistance and increased immunodeficiency (e.g., in AIDS and organ transplantation) have been increasing [6, 7].

The following route of TB transmission to the pancreas has been described. As a part of systemic miliary TB, spread to the pancreas can occur from retroperitoneal lymph nodes, and localized pancreatic TB can be caused by the movement of intestinal mycobacterium TB through the duodenal papilla. In the present case, the patient had tuberculous peritonitis but no signs of pulmonary TB. Therefore, pancreatic TB in this case may have been caused by a TB infection in the abdominal cavity.

There are no specific clinical symptoms or signs of pancreatic TB [8, 9]. Some patients with pancreatic TB may have a history of pulmonary, abdominal, or other TB types. However, less than half of patients have a history of TB or exhibit evidence of pulmonary TB by chest X-ray [8]. Pancreatic TB can cause chronic abdominal inflammation, leading to abdominal pain, epigastric discomfort, nausea, and radiative pain. In some patients with pancreatic TB, fusion block can be achieved by abdominal palpation. Pancreatic TB involving biliary and peripancreatic vessels can cause obstructive jaundice or portal hypertension, whereas pancreatic TB involving the stomach and duodenum can cause abdominal pain and acid regurgitation. Occasionally, pancreatic TB can also cause acute or chronic pancreatitis [10].

The qualitative diagnosis of pancreatic TB is difficult. Misdiagnosis may be related to the following factors: (1) the low incidence of pancreatic TB, the lack of specific clinical manifestations, and a clinician's lack of relevant knowledge; (2) the low clinical significance of general laboratory and biochemical/immunological examinations; and (3) the difficulty in diagnosing pancreatic TB by imaging, as patients may present with masses, cystic lesions, or abscesses as well as mass lesions in most cases of pancreatic carcinoma. The diagnosis of pancreatic TB eventually relies on pathological or microbiological results, including cytology, histology, acid-fast staining, TB culture, and PCR. The main methods for obtaining the required material include ultrasound-guided fine-needle aspiration, ultrasound- or CT-guided percutaneous fine-needle aspiration, and direct tissue excision (via open or laparoscopic surgery). Xia et al. [9] reported that 12 out of 16 patients underwent open surgery. Yan et al. [11] reported that 12 out of 13 cases required laparotomy. If histopathology indicates granulomatous lesions and caseous necrosis (epithelial cells and Langerhans giant cells are identified by cytological examination), pancreatic TB should be highly suspected. Positive acid-fast staining and TB culture results can support the diagnosis of pancreatic TB. However, the sensitivity of acid-fast staining is only 20–40% [12], and some patients will exhibit false-negative pulmonary TB results [7]. Additionally, the results report takes several weeks to obtain [13]. As a rapid and accurate diagnostic method, PCR has been applied in the diagnosis of TB with a sensitivity of 64% [14]. Of note, tumor marker CA-125 elevation does not exclude pancreatic TB. CA-125 is a homogeneous, high-molecular-weight glycoprotein that originates from the embryonic body cavity epithelium of various tissues. CA-125 has been widely recognized as a tumor marker, especially in ovarian cancer. However, this marker has no tissue or tumor specificity. Elevated levels of CA-125 may also occur in tuberculous peritonitis [15].

For patients with a definite diagnosis of pancreatic TB, regular antitubercular therapy for greater than 6 months can achieve significant improvement [1]. If the pancreatic TB lesions are relatively large, the lesions can be removed, e.g., by abscess drainage. Shunt surgery should be performed in patients with intestinal or biliary obstruction. Antitubercular therapy should be continued in patients with pancreatic TB.

## 4. Conclusions

Pancreatic TB can occur in children. It should be suspected when clinicians encounter pancreatic lesions despite the fact that elevated tumor markers, such as CA-125, may indicate malignancy. Even if both anti-acid stains smear and anti-TB antibody are negative, the diagnosis of pancreatic TB still cannot be completely excluded. Laparoscopy combined with peritoneal biopsy and PCR provides a novel method to confirm the diagnosis.
